# Quantitation of free glycation compounds in saliva

**DOI:** 10.1371/journal.pone.0220208

**Published:** 2019-09-18

**Authors:** Friederike Manig, Michael Hellwig, Franziska Pietz, Thomas Henle

**Affiliations:** Chair of Food Chemistry, Technische Universität Dresden, Dresden, Germany; King Abdulaziz University, SAUDI ARABIA

## Abstract

In the course of the Maillard reaction, which occurs during heating of food but also under physiological condition, a broad spectrum of reaction products is formed. Among them, the advanced glycation endproducts (AGEs) N^ε^-carboxymethyllysine (CML), pyrraline (Pyr), methylglyoxal-derived hydroimidazolone 1 (MG-H1) and N^ε^-carboxyethyllysine (CEL) are the quantitatively dominating compounds during later reaction stages. Those dietary glycation compounds are under discussion as to be associated with chronic inflammation and the pathophysiological consequences of diseases such as diabetes. In the present study, the concentration of individual glycation compounds in saliva was monitored for the first time and related to their dietary uptake. Fasting saliva of 33 metabolically healthy subjects was analyzed with HPLC-MS/MS. The observed levels of individual glycation compounds ranged from 0.5 to 55.2 ng/ml and differed both intra- and interindividually. Patterns did not correlate with subject-related features such as vegetarianism or sports activities, indicating that dietary intake may play an important role. Therefore, six volunteers were asked to eat a raw food diet free of glycation compounds for two days. Within two days, salivary Pyr was lowered from median 1.7 ng/ml to a minimum level below the limit of detection, and MG-H1 decreased from 3.6 to 1.7 ng/ml in in a time-dependent manner after two days. Salivary CML and CEL concentrations were not affected. Therefore, measuring Pyr and MG-H1 in saliva is a suitable diagnostic tool to monitor the dietary intake and metabolic transit of glycation compounds present in heated foods.

## Introduction

Nucleophilic sites of peptide-bound amino acids are prone to posttranslational protein modification reactions by reducing sugars and dicarbonyl compounds during the Maillard reaction (MR). The different stages of the MR take place during heating, storage and processing of food. Up to now, several free or protein-bound Maillard reaction products (MRPs) have been identified and quantitated in food and biological matrices. The early-stage Amadori product (AP) N^ε^-fructosyllysine (FruLys) is well characterized and used as a marker for the degree of low-temperature treatment [1,2]. The second stage of the MR is characterized by the formation of 1,2-dicarbonyl compounds such as methylglyoxal (MGO), glyoxal or 3-deoxyglucosone (3-DG), resulting from fragmentation reactions. Reaction products from later stages of the MR are known as “advanced glycation endproducts” (AGEs) [3,4]. The well-characterized N^ε^-carboxymethyllysine (CML) [5] derives from an intramolecular Cannizzaro reaction of the ε-amino group of lysine with glyoxal [6]. Depending on (physiological) reaction conditions, CML may also arise from an oxidative cleavage [6] or via the Namiki pathway [7]. In correspondence to CML, N^ε^-carboxyethyllysine (CEL) derives from MGO and lysine [8]. Pyrraline is formed during the reaction of lysine with 3-DG [9,10] predominantly during intense heating or long-term storage of foods. Beside lysine, the side chain of arginine is also prone for modifications during MR. The most prominent group of arginine MRPs are the hydroimidazolones, with methylglyoxal-derived hydroimidazolone 1 (MG-H1) being the most important in food and physiological systems [11,12]. The degree of modification and product range of the MR depend on the pH value of the food matrix since the pH value is known to modify e.g. the proportion of sugars in open-chain form, the formation of glucose degradation products and the reactivity of amino compounds [13]. Dietary AGEs are discussed as risk factors for diseases, such as diabetes and diabetic angiopathy [14–16]. Although this proposition is currently not yet verified, books available for the general public offer dietary recommendations on a diet low in MRPs [17]. Suchlike publications imply the emergent necessity of an easy tool to estimate the daily intake as well as the metabolic transit of alimentary MRPs through the human body.

The daily intake of MRPs was calculated after the comprehensive analysis of different food items with 3.1 ± 1.0 mg CML (~ 0.015 mmol), 2.3 ± 0.8 mg CEL (~ 0.01 mmol) and 21.7 ± 6.7 mg MG-H1 (~ 0.1 mmol) [18]. Pyr as the most important dietary MRP was estimated with an intake of 20 to 40 mg (~ 0.08–0.16 mmol) [19]. After ingestion, glycated proteins undergo proteolytic cleavage [20–22]. The translocation of the resulting peptides and amino acids depends on the chemical nature of the MRP. *In vitro* flux studies on Caco-2 cells suggested a transfer through the basolateral membrane for MRPs with an unpolar side chain such as Pyr, but not for molecules with a charged side chain such as CML [23,24]. In studies with human volunteers, a low recovery of FruLys in urine and faeces, but a high recovery of Pyr in urine was found, indicating the absorption of Pyr but not of FruLys [25–27]. A broad range of free MRPs such as CML, CEL, MG-H1 etc. were quantitated in human plasma in a nanomolar range [11].

However, sampling of plasma is invasive and requires well-trained medical staff. A method for high throughput-sampling would be preferable for diagnostic reasons. Saliva reflects a large amount of substances found in blood, that can be monitored for several purposes: hormones, pharmaceutical drugs, antibodies, carbohydrates, amino acids, tumor DNA, caffeine and many more [28–34]. The suitability of saliva as a diagnostic tool has been shown and is under current investigation for the early diagnosis of diseases, and therefore saliva is widely accepted as biological fluid for clinical diagnostics [32–35]. Furthermore, saliva allows a simple, painless, fast, permanently available, cheap and non-invasive sampling [36,37]. For a long time the salivary free amino acid profile has been under investigation as a diagnostic tool for phenylketonuria [38] and was suggested for the early diagnosis of cancer such as breast cancer [35] and oral squamous cell carcinoma [39]. The early diagnosis for an early detection of cancer was also suggested by different tumor-associated biomarkers such as mRNA, microRNA, lncRNA and proteins for IL-8, CD44, MMP-1 and MMP-3 [40,41]. Additionally, the monitoring of a chronic kidney disease via the salivary creatinine and urea levels was tested and allows an early diagnosis in children and adults [42–45].

In principle, amino acids can be quantitated in sweat and saliva [35,46,47]. This raises the question whether MRPs are detectable in human saliva or not. The aim of this study was to set up an LC-MS/MS multimethod and to survey the salivary “AGEome” regarding the AGEs CML, CEL, MG-H1, Pyr as well as the AP FruLys with a homogenous group of subjects.

## Material and methods

### Chemicals

LC-MS grade acetonitrile and HPLC grade methanol were purchased from VWR Prolabo (Darmstadt, Germany). Double distilled water (Bi 18E double distillation system, QCS, Maintal, Germany) was used for solvents for LC-MS analysis and nonafluoropentanoic acid (NFPA) was from Sigma-Aldrich (Steinheim, Germany). Reference material for calibration was synthesized as described before: N^ε^-fructosyllysine [48], pyrraline [24,49], CML [23], CEL [23] and MG-H1 [23]. Stable isotope labelled internal standards for HPLC-MS/MS analysis were synthesized in the same manner but using [^13^C_6_,^15^N_2_]lysine ([^13^C_6_,^15^N_2_]Pyr) and [^13^C_6_]arginine ([^13^C_6_]MG-H1) instead of the unlabeled compounds. [^13^C_3_]CEL was synthesized as described below. [^2^H_2_]CML was obtained from PolyPeptide (Strasbourg, France), [^13^C_6_,^15^N_2_]lysine and [^13^C_3_]sodium pyruvate from Campro (Berlin, Germany) and [^13^C_6_]arginine from Eurisotop (Saarbrücken, Germany). Purification was performed via semi-preparative ion-exchange chromatography and purity and identity of the products was evaluated with nuclear magnetic resonance spectroscopy, mass spectrometry, and amino acid analysis.

### Synthesis of [^13^C_3_]N^ε^-carboxyethyllysine

Based on a literature method [50], 61.5 mg (0.25 mmol) N^α^-Boc-lysine and 55.3 mg (0.5 mmol) [^13^C_3_]sodium pyruvate were dissolved in 5 mL 0.1 M sodium carbonate buffer, pH 10.0, and 200 mg sodium cyanoborohydride was added. The mixture was incubated at 37 °C for 3 days. In order to remove the Boc protecting group and excess cyanide, the mixture was diluted to 50 mL with water and incubated overnight in the presence of 80 mL of the strongly acidic cation exchange resin Lewatit S100 that had been equilibrated previously with 250 mL of 6 M HCl and 250 mL of water. Products bound to the resin were eluted with 300 mL 6 M HCl. The eluate was evaporated to dryness using a rotary evaporator, and the residue was taken up in 30 mL of 0.01 M HCl (pH = 2.3 after dissolution). This solution was applied to a column filled with the strongly acidic cation exchange resin DOWEX 50 WX-8 (30 mL) that had been equilibrated with 100 mL 6 M HCl, 100 mL water, and 100 mL 0.01 M HCl. Elution was performed first with 30 mL of 0.01 M HCl, then with 250 mL 1 M HCl, and, lastly, with 400 mL 1.5 M HCl. Fractions of 6 mL were collected by use of a fraction collector (BioRad). Fractions containing target substances were identified by spotting 1 μL of each fraction on TLC plates and spraying with a mixture of ninhydrin, acetic acid, and water (0.1/3/100, w/v/v). [^13^C_3_]CEL was found to elute between 230 and 300 mL of 1.5 M HCl. The respective fractions were pooled and evaporated to dryness. The residue was taken up in water and lyophilized to yield [^13^C_3_]CEL as a white solid.

Analytical data: HPLC-MS/MS: t_R_, 8.4 min; fragmentation (100 V, 10 eV) of [M + H]^+^ (*m/z* 222), 130 (100), 84 (91), 222 (24), 175 (5), 159 (4). Content = 76.2%, based on calibration with the unlabeled standard. Yield: 43.5 mg (59.7%).

### Study design

The study has been approved by the Ethics Committee of Technische Universität Dresden, Germany (reference: AZ 439112017). Written consent was obtained by each study participant. Altogether, the study included 55 participants, from which 22 subjects had to be excluded mostly due to low amounts of saliva. Fasting levels of salivary MRPs were analyzed in duplicate in samples from 33 metabolically healthy subjects. Saliva was sampled daily before breakfast. To investigate a putative dietary impact on salivary MRP levels, six subjects were asked to eat unheated food virtually free of MRPs [51] (mainly vegetables, fruits, unroasted nuts) for two days. Sampling was performed on day one until day three in the morning. The same group of subjects collected samples during another three days while eating their habitual diets including MRP-rich food.

### Sampling

Fasting saliva collection was performed at 8 am with Salivettes (Sarstedt, Germany). The manufacturer’s protocol for sampling was adapted to use Salivettes for saliva collection without stimulation. The subjects were asked to brush the teeth without tooth paste, rinse the mouth with water and wait for five minutes. Salivettes were placed in the middle of the tongue. The subjects were asked not to move the tongue within three minutes of saliva collection.

### Preparation of saliva samples for MRP analysis

Saliva was isolated from Salivettes by centrifugation (2 min, 2500 g). 500 μl saliva was mixed with10 μl internal standard solution as well as 490 μl of icecold acetonitril/methanol (70/30, v/v). After 10 min at 4 °C, the tubes were centrifuged (10000 g, 10 min), the supernatant was evaporated under nitrogen to dryness and the remnant was redissolved in 90 μL 20 mM NFPA. Samples were usually analyzed in duplicate. Double distilled water was used as basis for blanks. Blanks were treated like samples during sample processing. The solutions for external calibration of CML, CEL, MG-H1, Pyr, FruLys as well as Arg and Lys with each of the corresponding isotopologue internal standard were evaporated under nitrogen to dryness as well and resolved in 20 mM NFPA. Calibration curve for MRP was linear between 0.003–0.15 ng/ml and for amino acids between 0.1–150 μg/ml.

### High-pressure liquid chromatography with tandem mass spectrometric detection (HPLC-MS/MS)

Free MRPs were quantitated on an HPLC-MS/MS system consisting of a binary pump (G1312A), an online degasser (G1379B), an autosampler (G1329A), a column thermostat (G1316A), a diode array detector (G1315D), and a triple-quadrupole mass spectrometer (G6410A; all from Agilent Technologies, Böblingen, Germany). Nitrogen was utilized as nebulizing gas at the ESI source with a gas flow of 11 L/min, a gas temperature of 350 °C and a nebulizer pressure of 35 psi, and the capillary voltage was at 4000 V. Samples were run on a Phenomenex Kinetex C-18 column (50 x 2.1 mm, 1.7 μm, 100 Å) and an injection volume of 5 μL was used for chromatographic separation. Solvent A consisted of 10 mM NFPA in water, solvent B was 10 mM NFPA in acetonitrile. A gradient (0 min, 5% B; 10 min, 32% B; 11 min, 85% B; 14 min, 85% B; 15 min, 5% B) with a flow rate of 0.25 mL/min was used. For data aquisition the software Mass Hunter B.02.00 (Agilent) was used. Quantitation was performed using the MRM mode with ion transitions shown in Table 1. All samples were analyzed in duplicate.

**Table 1:** Parameters for mass spectrometric detection of (glycated) amino acids after chromatographic separation.

target analyte	precursor ion [m/z]	product ion [m/z]	dwell time [ms]	fragmentor voltage [V]	collision energy [eV]
**FruLys (^13^C_6_,^15^N_2_)**	317	233	70	70	13
**FruLys (^13^C_6_,^15^N_2_)**	317	129	70	70	15
**FruLys**	309	84	100	120	30
**FruLys**	309	225	100	120	10
**Pyr (^13^C_6_,^15^N_2_)**	263	182	100	75	5
**Pyr (^13^C_6_,^15^N_2_)**	263	153	100	75	20
**Pyr**	255	175	100	80	10
**Pyr**	255	148	100	80	20
**MG-H1 (^13^C_6_)**	235	74	100	75	20
**MG-H1 (^13^C_6_)**	235	115	100	75	10
**MG-H1**	229	114	100	120	10
**MG-H1**	229	166	100	120	10
**CEL (^13^C_3_)**	222	84	100	90	20
**CEL (^13^C_3_)**	222	130	100	90	10
**CEL**	219	130	100	100	10
**CEL**	219	84	100	100	20
**CML (^2^H_2_)**	207	84	100	90	20
**CML (^2^H_2_)**	207	130	100	90	10
**CML**	205	84	100	100	20
**CML**	205	130	100	100	10
**Arg (^13^C_6_)**	181	74	70	82	20
**Arg (^13^C_6_)**	181	61	70	82	10
**Arg**	175	70	70	90	20
**Arg**	175	60	70	90	10
**Lys (^13^C_6_,^15^N_2_)**	155	90	70	80	13
**Lys (^13^C_6_,^15^N_2_)**	155	137	70	80	3
**Lys**	147	84	70	75	10
**Lys**	147	130	70	75	10

### Statistics and method validation

The analysis of recovery was performed by spiking pooled saliva samples with known concentrations of analytes (standard addition method). Additionally, recovery rates for Salivettes were evaluated by adding a defined standard solution to Salivettes, incubation for 1 h at room temperature and sample preparation following the protocol stated above (*n* = 3). The precision of sample processing was determined by six-fold sample preparation and represents the intraindividual sample preparation variance. The precision of the LC-MS/MS method used during this study was calculated from six injections by using the coefficient of variation. Statistical analyses (correlation analysis: Spearman, test on normal distribution: Kolmogorov-Smirnov, One-Way-ANOVA after logarithmic data transformation) were performed by using the software OriginPro 9. Box plots were plotted with the same software. The box contains 50% of data and includes the mean (demonstrated with the square in the box) and the median (demonstrated with the line in the box). The length of the box corresponds to the interquartile range and is equivalent with the variation of the data. The whiskers within plots show a range of 1.5-fold values of the interquartile range. Outliers are indicated by an asterisk. A p-value < 0.05 was considered as statistically significant.

## Results

### LC-MS/MS quantitation of individual MRPs in saliva

Saliva has been valued as a diagnostic tool for several years [32,34,36]. To our knowledge, the identification of free individual glycation compounds in saliva were not part of any study before. Thus, the following study focused on the development of an LC-MS/MS method to analyze the levels of the prominent MRPs CML, CEL, MG-H1, Pyr and FruLys in saliva.

**Fig 1:**
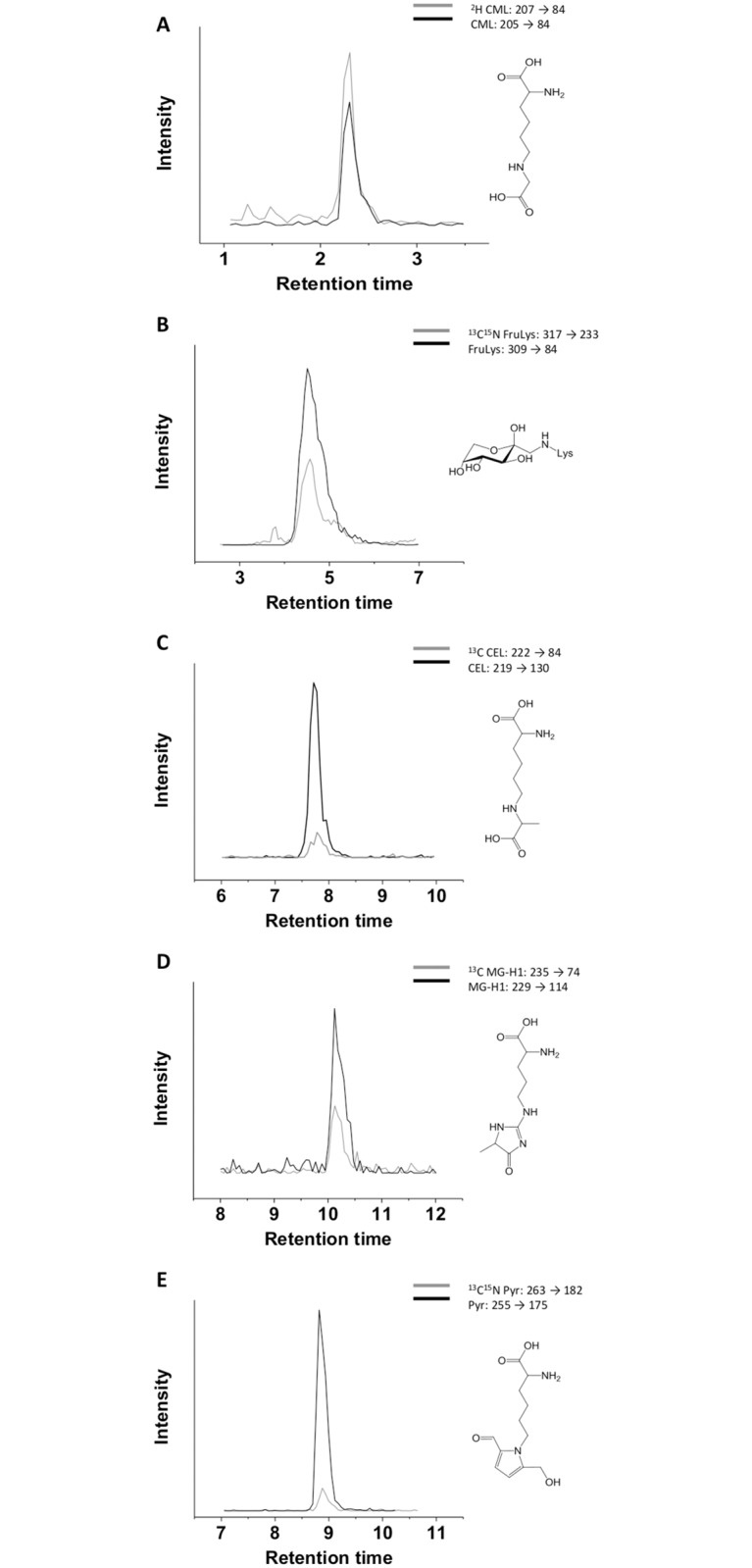
Chromatograms of the free MRPs A) CML, N^ε^-carboxymethyllysine; B) FruLys, N^ε^-fructosyllysine; C) CEL, N^ε^-carboxyethyllysine; D) MG-H1, methylglyoxal-derived hydroimidazolone 1 and E) Pyr, pyrraline in saliva. Each single analyte (grey) was analyzed in accordance with the corresponding isotopologue internal standard (black) by specific MRM transitions.

The sampling and analysis procedure had to cover three main requirements to allow a high sample throughput to gain reliable data: (i) an easy and reproducible saliva sampling protocol for untrained subjects, (ii) a sample preparation with acceptable efforts and costs, and (iii) a short run time of the LC-MS/MS method. To meet these challenges, sampling was performed with Salivettes and saliva was pre-concentrated after deproteinization. For the simultaneous quantitation of individual MRPs in saliva, an in-house method [52] was optimized for pre-concentrated saliva samples rich in electrolytes. The peak shape of the analytes suffered from time to time from secondary retention effects induced by salivary electrolytes, causing a basal peak broadening especially for FruLys. Furthermore, the electrolyte-rich matrix was found to cause peak shifts in retention time as it was often observed for CEL and CML. To solve these problems, product ion spectra obtained for saliva samples were compared with those identified after injection of standard solutions and an MRM method with distinctive transitions was used. Measurements were performed as stable isotope dilution assay for each analyte of interest to prevent matrix effects. The chromatographic separation of MRPs in saliva is shown in Fig 1 and was achieved by using an eluent system with nonafluorpentanoic acid as ion pair reagent as described earlier [52].

The calculation of the limit of detection (LOD) and limit of quantitation (LOQ) were based on the calibration curve method by using the calibration curve for each analyte. The concentration range covered with the calibration gave a linear signal response with coefficients of determination between 0.980 (MG-H1) and 0.999 (other analytes), respectively. Validation data are listed in Table 2. The respective LOD and LOQ are in agreement with data published earlier [11]. Recovery rates between 72.7 and 110.3% for the analysis with Salivettes were calculated. During our study we determined that the cotton of Salivettes retains up to 27% of the analyte in the case of FruLys or even led to higher yields of analytes (CEL) of the MRPs in saliva, most likely due to enrichment. The repeatability for LC-MS measurements was found to vary between 0.8% (Pyr) and 14.7% (MG-H1), repeatabilities for sample preparation between 1.4% and 16.3% were calculated.

**Table 2:** Performance and validation parameters of the LC-MS/MS method for the quantitation of (glycated) amino acids in saliva.

	FruLys	Pyr	MG-H1	CEL	CML
**Standard method parameters**					
**recovery [%]**	96.8	90.7	99.2	95.8	92.0
**Salivette recovery [%]**	72.7	100.6	82.7	110.3	85.8
**LOD [ng/Injection]**	0.02	0.05	0.04	0.02	0.03
**LOQ [ng/Injection]**	0.09	0.22	0.20	0.11	0.14
**Repeatability of LC-MS measurements**					
**Mean [ng/ml]**	7.4	1.1	2.8	1.8	8.5
**SD [ng/ml]**	0.4	0.0	0.4	0.2	1.0
**CV [%]**	5.8	0.8	14.7	9.7	12.1
**Repeatability of sample preparation**					
**Mean [ng/ml]**	7.7	1.1	2.4	1.8	7.2
**SD [ng/ml]**	0.3	0.1	0.4	0.1	0.6
**CV [%]**	4.3	1.4	16.3	4.5	8.7

### Interindividual variation of MRP levels in saliva

**Fig 2:**
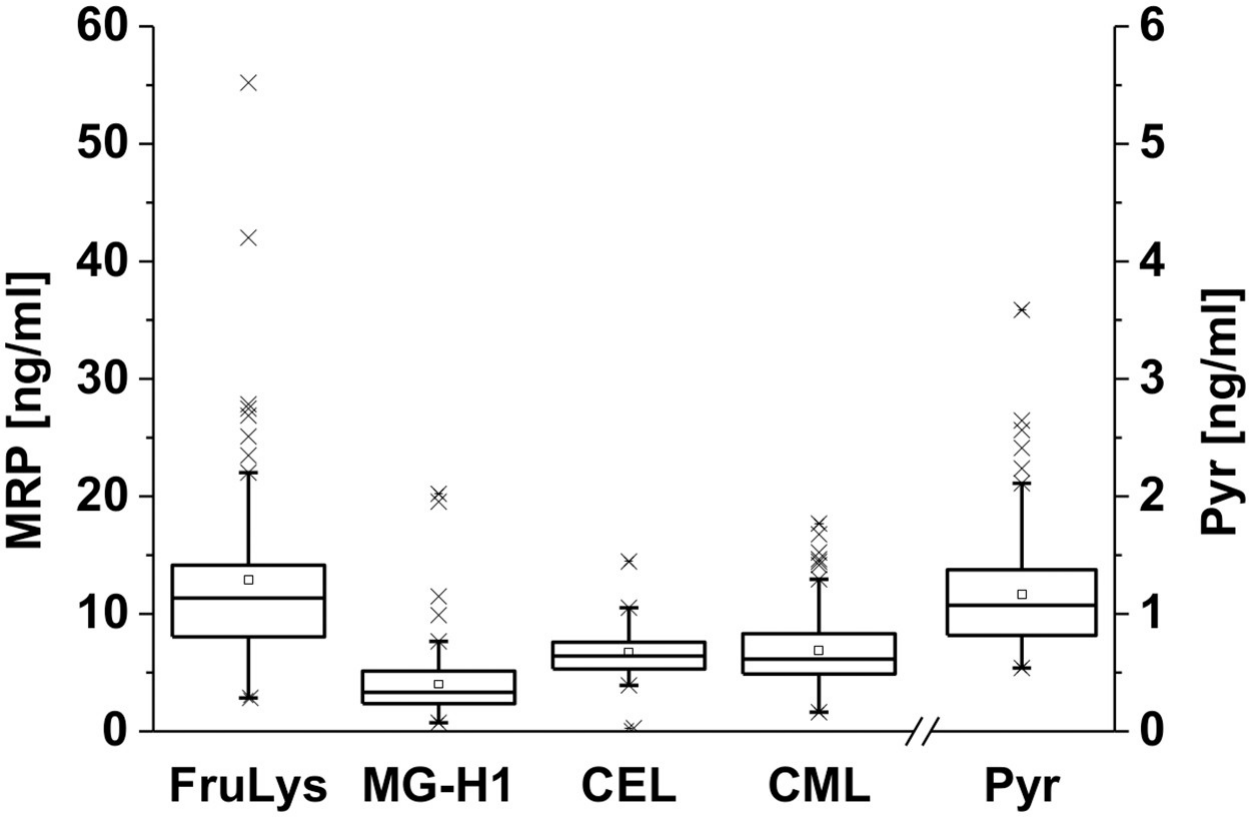
Concentrations of free MRPs in pre-concentrated saliva samples from 33 subjects on three consecutive days. The square indicates the mean, the line indicates the median. CEL was detected in 26/99 samples; presented data refer to samples with a CEL peak ratio S/N > 3.

The Maillard reaction products FruLys, Pyr, MG-H1, CML and CEL were quantitated in saliva samples of 33 subjects. The concentration ranges for the MRPs are shown in Fig 2. The mean concentrations of the free MRPs investigated differed significantly (except CEL/MG-H1 and CEL/CML). FruLys concentrations ranged from 4.5–57.5 ng/ml, whereas a more narrow concentration range of 0.5–1.6 ng/ml was found for Pyr. For MG-H1, CEL and CML, similar concentration ranges (0.3–24.5 ng/mL) were measured. The precursor amino acids of MRPs analyzed in this study, lysine and arginine, were also quantitated. The observed concentrations of free arginine and lysine were 0.4 to 10.4 μg/ml and 0.6 to 15.5 μg/ml, respectively, and are in agreement with data published by other authors [35,47,53]. Data for mean and median of each analyte as well as selected amino acids are documented in S1 Table (see supplement). Each participant of the study was asked to provide detailed information about the amount and components of meals, smoking behavior, blood group, sports activities and vegetarianism. No significant differences were found between MRP concentrations in saliva of people with different blood groups, vegetarians and omnivores as well as people with high, low or moderate sports activities (S2 Table). The inquiry for the amount of sports activities was crucial since physical activity is regarded as an influencing factor of endogenous dicarbonyl and consecutive MRP formation [54]. Vegetarians also showed no differences in salivary concentrations of the investigated analytes when compared to omnivores. Vegetarian food does not necessarily contain lower amounts of glycation products, because meat substitutes such as tofu or seitan are also heated during cooking and thus may represent a dietary source of MRPs. Furthermore, bakery products as the main MRP-providing food source was observed to be consumed in the same order of magnitude by both vegetarians and omnivores. The smoking behavior may also influence the salivary MRP concentration due to the formation of dicarbonyl compounds during smoking [55]. A correlation analysis of smoking behavior with salivary MRP concentrations was not successful due to low participation of subjects smoking ≥ 5 cigarettes/day and due to low sample amounts. In particular, the delivery of a low sample amount was frequently the case for smoking subjects and elderly people. Thus, the influence of smoking and age on salivary MRP concentrations has to be further elucidated.

For further assessment of relations between the occurrence of MRPs, correlation analysis between the individual analytes in saliva was performed according to the models of Spearman (S3 Table). Concerning the MRPs, a moderate correlation was observed for Pyr and CML with FruLys as well as Lys and FruLys. Interestingly, this is also the case for Lys and Arg, showing moderate correlations to most investigated MRPs. This observation may be attributable to similar transport mechanisms of Arg and Lys from blood to saliva, what may be also the case for the MRPs originating from Arg and Lys. CEL is the only analyte showing no correlation to any other compound analyzed in this study.

### Intraindividual variation of MRP levels in saliva

To assess intraindividual variations between different days of salivary MRP levels of each subject, the subjects were asked to give samples from three consecutive days. Samples were measured as stated above, and the intraindividual variation was calculated as coefficient of variation. The analysis revealed high intraindividual variations for FruLys, MG-H1, lysine, and arginine, ranging between 5 and 80–90% (see Table 3 and S1 Fig). Remarkably, FruLys showed high and Pyr low inter- and intraindividual variations. The interindividual variations overlap the intraindividual variations. A profile of the investigated MRPs from different subjects is given in S1 Fig. The intraindividual variations may be explained by alimentary MRP intake, smoking behavior, individual metabolic or even pathophysiologic characteristics. It was described for several substances that saliva mirrors blood concentrations [31,32,44,56]. This assumption is supported by two arguments: A strong age-dependent increase of MRPs in biological fluids was neither seen in this study nor reported to be pronounced in the literature [57].

**Table 3:** Intraindividual variations of fasting salivary levels of selected (glycated) amino acids.

	FruLys	Pyr	MG-H1	CEL	CML
Mean [%]	32.7	21.7	34.5	18.9	29.9
Median [%]	24.1	19.4	30.2	21.1	26.7
Variation [%]	4.9–95.5	3.0–38.7	8.9–87.8	8.7–32.7	5.9–82.2

FruLys, CML, CEL, MG-H1 and Pyr as well as arginine and lysine were analyzed in saliva from 33 subjects (CEL: 6 subjects) collected on three consecutive days in the morning. Intraindividual variation data given as percentage of the coefficient of variance of each subject within three days.

### Preliminary study concerning a dietary impact

**Fig 3:**
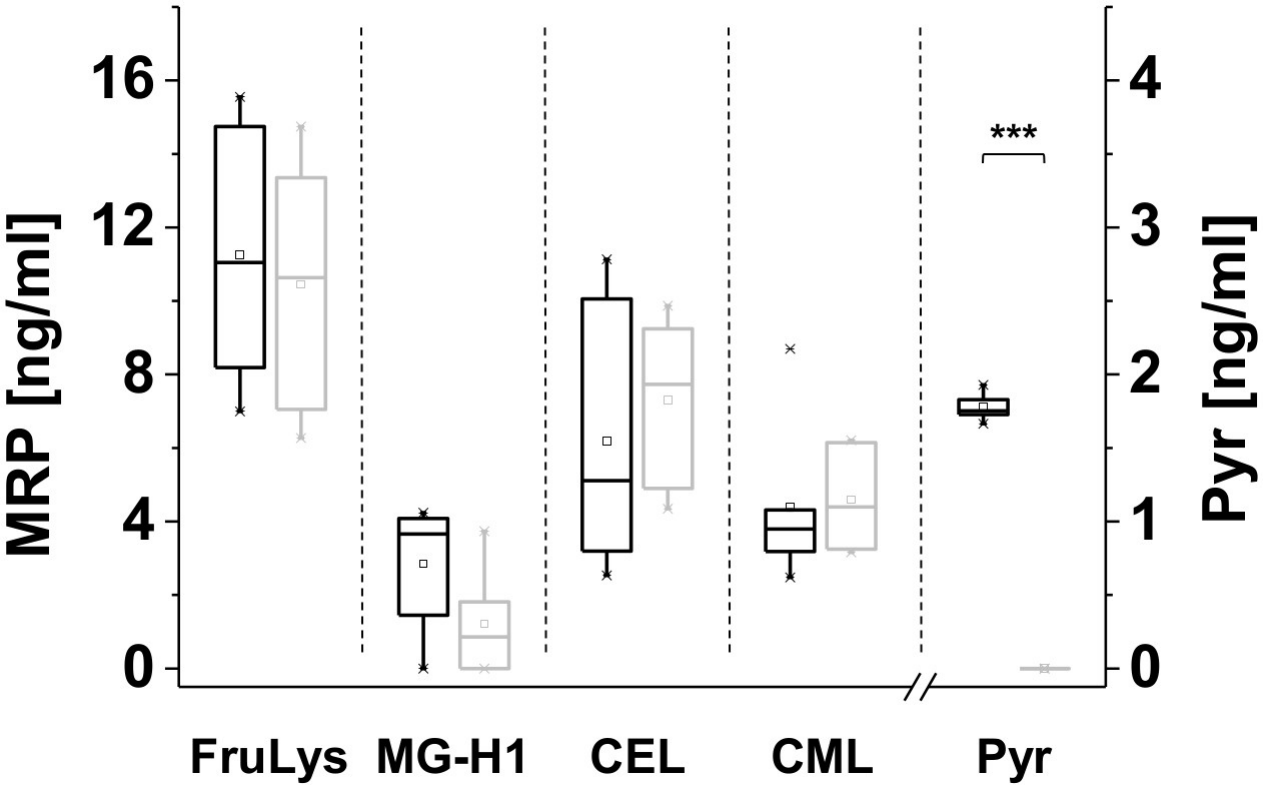
Investigation of an alimentary impact on salivary Maillard reaction compounds. Salivary MRP levels of six subjects following a diet low in Maillard reaction products (MRP) from day 1 (black, left, usual diet) to day 3 (gray, right, two days low MRP diet), presented as box plot. Samples were analyzed in duplicate. Significance: *** significantly differed with a p-value < 0.005.

The salivary levels of the investigated analytes showed high inter- and intraindividual variations, that could not be explained by any of the personal parameters asked in the questionnaire. Evaluation of meals ingested by the participants as reported in the questionnaire implied a dietary influence on salivary MRP levels. In order to analyze whether the concentrations of individual MRPs in saliva depend on the dietary intake, a group of volunteers homogenous in age and smoking behavior (light or non-smoking) was asked to eat raw or unheated food for two days. The supply with protein was ensured by eating unroasted, unheated nuts (cashews were excluded because cashews may be heated during peeling and processing) and approx. < 20 g raw milk cheese low in MRPs per day. On the first day of the diet, salivary MRP levels were consistent with the results from the survey study. After only two days, Pyr was no more detectable in the samples or only in low amounts, respectively (Fig 3). MG-H1 also decreased in a time-dependent manner. Salivary concentrations of CML and CEL did not decrease. For CEL, a slight increase could be observed. No clear statement can be given about a dietary impact on salivary FruLys levels, what has to be further elucidated. In conclusion, salivary Pyr and MG-H1 levels seem to be influenced by the diet.

## Discussion

Several invasive and non-invasive methods are available to determine the concentration of glycation products in body fluids and tissues [11,18,58–60]. While glycation products have often been measured in urine, saliva has not yet been investigated as a potential matrix for non-invasive quantitation of MRPs. In the present study, individual salivary MRPs were characterized for the first time. We focused on the analysis of free glycated amino acids because MRPs circulating in blood *in vivo* are associated with dietary ingested MRPs [27,61,62]. Based on literature surveys, the sampling procedure was optimized first. In the literature it is well described that that stimulation leads to changes in the composition as well as concentrations in saliva [60,61], e. g. for amino acids [44]. To avoid effects of stimulation, we decided to use Salivettes for sampling, but without chewing as suggested by the supplier. However, we found Salivettes to be the most preferred reliable saliva sampling technique for untrained subjects.

Recently, serum concentrations of diabetes type II—related MRPs were quantitated by a tandem mass spectrometric technique in non-diabetic subjects and found to be 17.5 ± 8.2 ng/ml for MG-H1, 9.8 ± 2.2 ng/ml for CEL and 15.1 ± 4.2 ng/ml for CML [59], which is consistent with former data from Thornalley and colleagues in 2003 [11] with plasma concentrations of CML (4.7 ± 1.6 ng/ml), CEL (7.6 ± 3.1 ng/ml) and MG-H1 (25.1 ± 10.5 ng/ml). Concentrations of individual MRPs in saliva in the present study were ranging from 0.5–65.8 ng/ml and were found to occur in the same order of magnitude as in plasma, serum and human milk [11,59,60,63]. This observation seems to be plausible as it is known that salivary analyte concentration reflects serum and plasma concentrations. We hypothesize that the MRPs found in saliva originate from blood. The transport of biomolecules into saliva occurs through diffusion, filtration or active transport mechanisms [34]. The type of MRP transport from blood to saliva remains to be elucidated.

Furthermore, the results imply no correlation between the salivary MRP concentration and any other parameter tested such as blood group or vegetarianism (S2 Table). The survey about salivary MRP levels of 33 metabolically healthy subjects does not allow a conclusion about the source of salivary MRPs. In principle, MRPs in saliva can be a result of a metabolic transit from diet as an exogenous source to saliva, but may also result from endogenous processes. Therefore, we implemented a preliminary study about a putative alimentary impact on salivary MRP levels and to learn about the metabolic transit of MRPs into saliva. As it was shown with metabolomics analysis, there is no consensus about an alimentary influence of the salivary composition in the literature [64,65]. In the same manner, the dietary influence on salivary concentrations of MRPs cannot be supposed to be generic: We are the first to show that the observed intraindividual variations of Pyr and MG-H1 in saliva are obviously associated with the dietary intake of the respective compounds but CML, CEL and FruLys are most likely not. The source of MRP levels in biological fluids has been of scientific interest for some years. The renal excretion of FruLys, analyzed as furosine, as well as Pyr and CML after a defined intake from the diet was investigated in a study with human volunteers [27]. In this study, 3% of dietary FruLys was recovered in the urine, implying low absorption—in opposite to Pyr, of which more than 50% of the ingested amount was recovered. Similarly, a high dietary impact on salivary Pyr levels appears likely: Pyr was found to pass the basal membrane in a Caco-2 cell model, whereas the permeability for CML and MG-H1 was impaired [23,24]. Furthermore, our results confirm the study of Förster et al. [27] where it was shown that urinary Pyr is almost completely of dietary origin. No dietary impact was observed for urinary CML [27]. Concerning CML, published data imply contradictory results. The metabolic transit of CML, CEL and MG-H1 from food to plasma and urine was recently published by Scheijen and colleagues [18]. The intake of the MRPs was calculated from questionnaires. An alimentary effect on plasma and urine levels was described for CML, CEL and MG-H1. The intake from the diet was estimated to 2–4 mg/d CML, 1.6–3.2 mg/d CEL and 15–29 mg/d MG-H1. The authors analyzed the following plasma levels of free MRPs, here calculated as ng per ml: CML 12–20 ng/ml, CEL 6.3–13.0 ng/ml and MG-H1 19.5–43.6 ng/ml independent of the study group. It was shown, that higher MRP intake was associated with higher plasma and urine levels of free, but not protein-bound MRPs. The most significant effect due to the diet was found for MG-H1.

In conclusion, saliva is a suitable matrix for the analysis of glycation compounds, in particular to address the question if dietary MRPs play a part in the MRP pool *in vivo*. Our results suggest that MRP levels in saliva may be useful biomarkers for the assessment of COPD [57,66], coronary artery calcification [58] and diabetes as the levels of some MRPs such as CEL, FruLys, MG-H1 and CML are higher in body fluids and proteins of diabetes patients [58,67]. However, there is still insufficient knowledge about individual chemical structures and/or profiles of MRPs associated with defined disease states especially at the onset of metabolic disorders. This knowledge is a prerequisite for the use as biomarker as well as knowledge about the concentration of MRPs at different disease states. Corresponding studies are currently underway in our laboratory.

## Supporting information

S1 Fig Intraindividual variation of Maillard reaction products in saliva. Monitoring of fasting salivary MRP profiles of 33 subjects different in age and sex each on three consecutive days.
(TIF)

S1 Table Individual fasting salivary levels of Maillard reaction products and selected amino acids in saliva. (DOCX)

S2 Table Correlation of subject characteristics with salivary MRP levels. (DOCX)

S3 Table Correlation analysis of individual salivary MRPs. (DOCX)

## Abbreviations

3-DG: 3-deoxyglucosone
AGE(s): advanced glycation endproduct(s)
AP(s): Amadori product(s)
Arg: arginine
CEL: N^ε^-carboxyethyllysine
CML: N^ε^-carboxymethyllysine
FruLys: N^ε^-fructosyllysine
HPLC: high pressure liquid chromatography
LC-MS: liquid chromatohraphy coupled with mass spectrometry
Lys: lysine
MG-H1: methylglyoxal-derived hydroimidazolone 1
MGO: methylglyoxal
MRP(s): Maillard reaction product(s)
NFPA: Nonafluoropentanoic acid
Pyr: Pyrraline
