# Supplementary material for: Quantitation of free glycation compounds in saliva
Source: PLoS One. 2019 Sep 18;14(9):e0220208. doi: 10.1371/journal.pone.0220208 (PMC6750567; doi:10.1371/journal.pone.0220208)
Supplement: S1 Table — (DOCX) [file pone.0220208.s002.docx]

**S1 Table: Individual fasting salivary levels of Maillard reaction products and selected amino acids in saliva.**

|  | **FruLys [ng/ml]** | **Pyr [ng/ml]** | **MG-H1 [ng/ml]** | **CEL [ng/ml]** | **CML [ng/ml]** | **Arg [µg/ml]** | **Lys [µg/ml]** |
| --- | --- | --- | --- | --- | --- | --- | --- |
| **Range** | 2.9 – 65.8 | 0.5 – 3.6 | 0.7 – 20.2 | n.d. – 14.5 | 1.6 – 24.2 | 0.4 – 10.4 | 0.6 – 15.5 |
| **Mean** | 13.8 | 1.2 | 4.2 | 6.7 | 7.1 | 2.5 | 3.7 |
| **1st quartile** | 8.4 | 0.8 | 2.4 | 5.2 | 4.9 | 1.4 | 1.8 |
| **Median** | 11.5 | 1.1 | 3.4 | 6.4 | 6.2 | 2.0 | 2.9 |
| **3rd quartile** | 14.4 | 1.4 | 5.2 | 7.8 | 8.6 | 3.3 | 4.5 |

FruLys, CML, CEL, MG-H1 and Pyr as well as arginine and lysine were analyzed in saliva from 33 subjects collected on three consecutive days in the morning. 1^st^ quartile = 25 %, 3^rd^ quartile = 75 %. CEL was detected in 26/99 samples; presented data refer to samples with a CEL peak ratio S/N > 3.
