# Supplementary material for: Quantitation of free glycation compounds in saliva
Source: PLoS One. 2019 Sep 18;14(9):e0220208. doi: 10.1371/journal.pone.0220208 (PMC6750567; doi:10.1371/journal.pone.0220208)
Supplement: S2 Table — (DOCX) [file pone.0220208.s003.docx]

**S2 Table: Correlation of subject characteristics with salivary MRP levels.**

| **Omnivore** | **FruLys** | **Pyr** | **MG-H1** | **CML** | **CEL** |
| --- | --- | --- | --- | --- | --- |
| **Bonferroni** | 1 | 1 | 1 | 1 | - |
| **Tukey** | 1 | 1 | 1 | 1 | - |
| **Bonferroni-Holm** | 0.99 | 1 | 0.99 | 1 | - |
| **Vegetarians** | **FruLys** | **Pyr** | **MG-H1** | **CML** | **CEL** |

| **Blood group** | **Bonnferoni** | **Tukey** | **Bonferroni-Holm** |
| --- | --- | --- | --- |
| **A – B** | 0.70 | 0.39 | 0.12 |
| **A – AB** | 1 | 0.98 | 0.20 |
| **B – AB** | 1 | 0.82 | 0.39 |
| **0 – A** | 1 | 0.58 | 0.58 |
| **0 – B** | 1 | 0.96 | 0.63 |
| **0 – AB** | 1 | 0.94 | 0.70 |
