# Supplementary material for: Quantitation of free glycation compounds in saliva
Source: PLoS One. 2019 Sep 18;14(9):e0220208. doi: 10.1371/journal.pone.0220208 (PMC6750567; doi:10.1371/journal.pone.0220208)
Supplement: S3 Table — (DOCX) [file pone.0220208.s004.docx]

**S3 Table: Correlation analysis of individual salivary MRPs.**

| **Spearman** | | **FruLys** | **Pyr** | **MG-H1** | **CEL** | **CML** | **Arg** |
| --- | --- | --- | --- | --- | --- | --- | --- |
| **FruLys** | r |  |  |  |  |  |  |
|  | p |  |  |  |  |  |  |
| **Pyr** | r | 0.669 |  |  |  |  |  |
|  | p | < 0.001 |  |  |  |  |  |
| **MG-H1** | r | 0.571 | 0.553 |  |  |  |  |
|  | p | < 0.001 | < 0.001 |  |  |  |  |
| **CEL** | r | 0.398 | 0.342 | 0.402 |  |  |  |
|  | p | 0.036 | 0.075 | 0.034 |  |  |  |
| **CML** | r | 0.412 | 0.356 | 0.575 | 0.265 |  |  |
|  | p | < 0.001 | < 0.001 | < 0.001 | 0.173 |  |  |
| **Arg** | r | 0.460 | 0.270 | 0.546 | 0.249 | 0.574 |  |
|  | p | < 0.001 | < 0.001 | < 0.001 | 0.201 | < 0.001 |  |
| **Lys** | r | 0.625 | 0.487 | 0.585 | 0.440 | 0.589 | 0.647 |
|  | p | < 0.001 | < 0.001 | < 0.001 | 0,019 | < 0.001 | < 0.001 |

Correlation between salivary Maillard reaction products and selected amino acids was performed with the Spearman model as non-parametric approach.
